# Patterns of Biomass and Carbon Distribution across a Chronosequence of Chinese Pine (*Pinus tabulaeformis*) Forests

**DOI:** 10.1371/journal.pone.0094966

**Published:** 2014-04-15

**Authors:** Jinlong Zhao, Fengfeng Kang, Luoxin Wang, Xiaowen Yu, Weihong Zhao, Xiaoshuai Song, Yanlei Zhang, Feng Chen, Yu Sun, Tengfei He, Hairong Han

**Affiliations:** College of Forestry, Beijing Forestry University, Beijing, China; DOE Pacific Northwest National Laboratory, United States of America

## Abstract

Patterns of biomass and carbon (C) storage distribution across Chinese pine (*Pinus tabulaeformis*) natural secondary forests are poorly documented. The objectives of this study were to examine the biomass and C pools of the major ecosystem components in a replicated age sequence of *P. tabulaeformis* secondary forest stands in Northern China. Within each stand, biomass of above- and belowground tree, understory (shrub and herb), and forest floor were determined from plot-level investigation and destructive sampling. Allometric equations using the diameter at breast height (*DBH*) were developed to quantify plant biomass. C stocks in the tree and understory biomass, forest floor, and mineral soil (0–100 cm) were estimated by analyzing the C concentration of each component. The results showed that the tree biomass of *P. tabulaeformis* stands was ranged from 123.8 Mg·ha^–1^ for the young stand to 344.8 Mg·ha^–1^ for the mature stand. The understory biomass ranged from 1.8 Mg·ha^–1^ in the middle-aged stand to 3.5 Mg·ha^–1^ in the young stand. Forest floor biomass increased steady with stand age, ranging from 14.9 to 23.0 Mg·ha^–1^. The highest mean C concentration across the chronosequence was found in tree branch while the lowest mean C concentration was found in forest floor. The observed C stock of the aboveground tree, shrub, forest floor, and mineral soil increased with increasing stand age, whereas the herb C stock showed a decreasing trend with a sigmoid pattern. The C stock of forest ecosystem in young, middle-aged, immature, and mature stands were 178.1, 236.3, 297.7, and 359.8 Mg C ha^–1^, respectively, greater than those under similar aged *P. tabulaeformis* forests in China. These results are likely to be integrated into further forest management plans and generalized in other contexts to evaluate C stocks at the regional scale.

## Introduction

Biomass and carbon (C) storage in forest ecosystems play dominant roles in global C cycle [1], [2], [3], [4], and serve as the most significant C sinks to reduce global warming [5]. This is largely due to their huge potential for sequestering carbon in vegetation and soil [6], [7], [8], [9], and interact with atmospheric processes through the absorption and respiration of CO_2_ [2], [10], [11]. As an important C pool in the terrestrial ecosystems, forests store more C than any other terrestrial ecosystems [8] and contain about 80% of all aboveground terrestrial C [12] and 40% of soil C [2], [13]. Therefore, a slight change of the C pool could have an important impact on global C balance. In the past few decades, many studies have estimated biomass or C storage in ecosystem components such as vegetation, forest floor and mineral soil [14], [15], [16], [17], [18]. However, considerable problems and uncertainties existed in these studies due to site-specific characteristics, and inconsistent methodologies and definitions [19]. Thus, there is a need for accurate information concerning the biomass and C storage in forest ecosystems to improve our understanding in processes and mechanisms of the global C cycle.

Generally speaking, stand development is bound up with C storage over the entire life cycle of forest ecosystems because tree growth rates vary greatly with stand age [20], [21]. Furthermore, stand age is one of the crucial factors affecting changes in C allocation among different forest ecosystem components such as the forest floor, soil, and coarse woody debris [15], [22], [23], [24], [25]. Moreover, it affects allometry, wood density and structure [26], [27], [28], which has a considerable impact on the quantitative analysis of forest biomass. To date, many studies based on age have reported that the biomass and C storage patterns in forest ecosystems [3], [4], [29]; however, few studies have examined biomass and C storage of *P. tabulaeformis* forests across a chronosequence, particularly in Northern China.

*P. tabulaeformis* is a geographically widely distributed native tree species that spans in Northern China from latitude 31°13′ N to 43°33′ N and from longitude 103°20′ E to 124°45′E. Its ability to grow in poor site conditions as well as regenerate naturally as a secondary succession pioneer species following disturbances has led to the tree species covering a total of 228.10×10^4^ ha [30] of forestland in China. Although biomass or C storage of this tree species in China have been quantified [31], [32], studies on the forest C stock and C allocation patterns along stand development are scarce [29]. Due to rapid land use changes, *P. tabulaeformis* and other secondary forests have been continuously increasing in their coverage in China. Secondary forests provide important ecosystem services, including erosion prevention, biodiversity maintenance, water conservation and watershed protection [33]. In recent years, many efforts have been made to understand the ecological processes of secondary forests [34], [35], [36]. However, there is still a lack of information on the biomass and C storage in *P. tabulaeformis* natural secondary forests in an age sequence,which is crucial for us to predict the responses of regional and global carbon balance to future climate change.

The objectives of this study were (1) to estimate the biomass of the ecosystem components across an age sequence of *P. tabulaeformis* stands, and (2) to assess the changes in the size and contribution of these C pools to total ecosystem C stock with increasing stand age.

## Materials and Methods

### Site description

**Figure 1:**
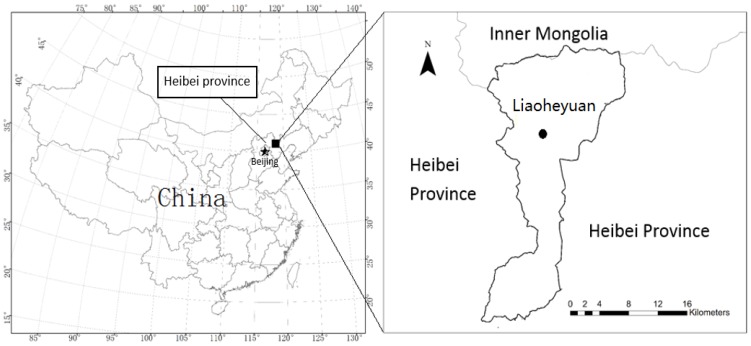
The location of Liaoheyuan, Hebei Province, China.

The study was conducted in Liaoheyuan (41°01′∼41°21′N, 118°22′∼118°37′E), located in Pingquan County, Hebei Province (Fig. 1) (Pingquan forestry bureau issued the permission to conduct this study for each location). Altitude ranges from 625 to 1738 m above sea level. This area is warm temperate to cold temperate transition zone, a semi-humid and semi-arid continental monsoon mountain climate with a humid and rainy summer and a cold and snowy winter. The mean annual temperature is 7.3°C (−10.8°C in January and 22.9°C in July, respectively), and the mean annual precipitation is 540 mm (most rainfall occurs from May to August). The annual frost-free period is 110–125 days, and annual mean total sunshine time is 2,000–2,900 h. The soil is a typical brown forest soil with a thickness of approximately 100 cm.

### Experimental design

Based on the “National Forest Resource Continuous Investigation Technical Regulations”[37], our P. tabulaeformis chronosequence (four age groups) includes twelve stands (three stands in each age group, and they were natural regeneration) with pure overstories, ≤30, 31–50, 51–60, and 61–80-year-old, representing the young, middle-aged, immature, and mature stages, respectively. Understory of all stands is dominated by Quercus liaotungensis, Quercus mongolica, Lespedeza bicolor, Spiraea trilobata, Rhamnus parvifolia, Corylus mandshurica, Rhododendron micranthum, Deutzia grandiflora, Prunus armeniaca, and herbs such as Carex rigescens, Saussurea nivea, Dianthus chinensis, Polygonatum odoratum, Dontostemon dentatus, Vicia unijuga,chinensis, and Goodyera schlechtendaliana.

Within each stand, three 20 m×30 m permanent plots (50 m apart each plot) were randomly set up in July 2012. Species identities, diameter at breast height (*DBH*), height and crown dimensions were documented for all trees within each plot. Detailed information for these forest stands is shown in Table 1.

**Table 1:** Characteristics of stands of *P. tabulaeformis* natural secondary forests.

Age groups	Stand age (years)	Location (lat, long)	Elevation (m)	Slope degree (°)	Stand density (stems·ha^–1^)	Bulk density* (g·cm^–3^)	*DBH* * (cm)	Height* (m)	Basal area of *DBH* (m^2^·ha^–1^)	Stand volume** (m^3^·ha^–1^)
Young	<30	41°18′N, 118°30′E	1097±6	27±2	2567±112	1.5±0.1	11.0±5.1	9.2±2.0	29.5	270.2
		41°19′N, 118°33′E	1094±5	29±2	2334±35	1.7±0.2	11.8±5.5	10.2±2.1	31.0	283.4
		41°18′N, 118°31′E	1058±10	30±1	1900±93	1.4±0.1	11.0±6.1	9.6±2.4	23.5	215.3
Middle-aged	31–50	41°17′N, 118°31′E	1008±8	30±2	1034±9	1.6±0.1	18.4±7.5	14.9±4.2	32.1	307.2
		41°18′N, 118°32′E	982±3	23±1	1050±28	1.6±0.1	18.5±5.2	15.8±2.0	30.4	291.5
		41°20′N, 118°34′E	985±2	25±1	1034±41	1.5±0.1	17.2±4.6	13.2±2.6	25.8	247.6
Immature	51–60	41°15′N, 118°31′E	1011±14	31±2	884±17	1.6±0.1	18.9±7.7	15.9±4.2	28.9	276.7
		41°17′N, 118°30′E	993±15	30±3	850±53	1.5±0.1	20.4±8.4	17.5±4.4	32.5	263.9
		41°19′N, 118°31′E	1018±9	31±1	867±22	1.4±0.1	20.0±5.3	17.0±2.1	29.2	237.3
Mature	61–80	41°18′N, 118°28′E	1066±18	29±2	917±80	1.6±0.1	23.3±7.1	20.9±3.7	42.5	419.3
		41°16′N, 118°31′E	1080±15	31±2	934±106	1.5±0.2	22.9±10.2	20.1±4.3	45.8	452.4
		41°20′N, 118°30′E	1089±10	23±1	717±37	1.5±0.2	23.1±12.5	19.8±4.9	38.5	379.9

^*^: stand mean ± within-stand deviation *(SD).*

^**^: Stand volume (*M*) and sample tree volume (*V*) was calculated by the following formula (1) and (2), respectively.

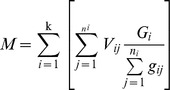
(1); 
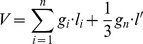
 (2)Where *M* is the stand volume (m3·ha–1), *n_i_* is the number of *i*–th class of the sample tree, *k* is graded series (*i*  = 1, 2,…, *k*), *G_i_* is the *i*–th class of basal area of *DBH* (m2·ha–1), *V_ij_* and *g_ij_* are the volume (m3) and basal area of *DBH* (m2) of *j*–th sample tree in *i*–th class, respectively. *V* is the sample tree volume (m^3^), *g_i_* is the central basal area (m2) of the *i*–th log, *l_i_* is the length (m) of the *i*–th log, *g_n_* is the last basal area (m2) at the top of tree, *l′*is the length (m) between the last basal area and the top of tree, *n* is the total number of logs.

### Field sampling and measurements

#### Tree and understory biomass estimation

This work was conducted based on Forestry Standards“Observation Methodology for Long-term Forest Ecosystem Research” of People's Republic of China [38].

In early August 2012, five *P. tabulaeformis* trees within representative stand-specific *DBH* range were selected and harvested destructively in each stand (The Pingquan forestry bureau issued the permission to conduct this study for each location). Trees were cut at the buttress near the ground surface. After the measurements of the diameters at *DBH* and at ground level (*D_0_*), and the total height (*H*), the tree (including the tree stem, branch, needle, and pine cone) was first cut open at 1.3 m. The top part (from 1.3 m to the tips) was then sectioned into 1 m (if *H*<15 m) or 2 m (if *H*>15 m) intervals using “stratified cutting method” [38]. All branches, needles and pine cones of each stem section were clipped from the tree stems and branches, respectively. The fresh mass of logs (sectioned tree stems) and canopy (branches, needles and pine cones separately) were measured in situ. Stem disks of approximately 5 cm thickness from the stump were cut out of each section, and separated into two subsamples: stem wood and stem bark. In each stand, roots of each sampled tree were excavated manually in 1 m depth within the radius of 1 m from the tree center, and sorted into five size classes: piles, coarse roots (with a diameter >5 cm), big roots (with a diameter between 2 and 5 cm), taproots (with a diameter between 1 and 2 cm) and fine roots (with a diameter <1 cm). Then the entire root system was washed lightly to remove soil particles, air dried, and weighed. Subsamples (stems, barks, branches, needles, pine cones, and roots) of each tree were brought back to the laboratory and oven dried at 80°C to constant weight for dry biomass determination.

Previous study demonstrated that *DBH*-based allometric equations [25] could be used to estimate tree biomass in an age sequence of pine forests. Cao et al. [29] also reported that tree components biomass of *P. tabulaeformis* forests in northern China were highly correlated with *DBH* (*R^2^* values were all over 96%). Using the same *DBH*-based allometric equations, we found they almost explained more than 90% of the variability in all components between the young and mature *P. tabulaeformis* stands in our study area (Table 2). Thus, these equations were used to estimating tree components biomass of the *P. tabulaeformis* forests.

**Table 2:** Parameters and statistics of biomass equations for different tree components in *P. tabulaeformis* forests.

Age groups	Components	*a*	*b*	*R*	*F* value	*S.E.E* a	*MSR^b^*	*P*
Young	Total tree	4.401	1.031	0.965	83.156	0.046	0.002	<0.01
	Aboveground	3.135	1.085	0.965	82.283	0.049	0.002	<0.01
	Tree stem	1.281	1.220	0.966	84.735	0.054	0.003	<0.01
	Stem wood	1.477	1.078	0.947	53.952	0.060	0.004	<0.01
	Stem bark	0.048	1.874	0.972	105.233	0.075	0.006	<0.01
	Branch	1.045	0.919	0.958	68.570	0.045	0.002	<0.01
	Needle	1.243	0.802	0.946	52.324	0.045	0.002	<0.01
	Pine cone	6E-05	3.587	0.942	48.760	0.210	0.044	<0.01
	Roots	1.450	0.800	0.926	37.480	0.053	0.003	<0.01
Middle-aged	Total tree	9.799	1.009	0.949	55.928	0.025	0.001	<0.01
	Aboveground	10.057	0.965	0.918	33.467	0.031	0.001	<0.05
	Tree stem	8.440	0.950	0.859	18.336	0.041	0.002	<0.05
	Stem wood	6.597	0.997	0.845	16.400	0.045	0.002	<0.05
	Stem bark	2.765	0.551	0.933	42.096	0.016	0.000	<0.01
	Branch	0.303	1.386	0.932	40.826	0.040	0.002	<0.01
	Needle	2.653	0.589	0.910	30.200	0.020	0.000	<0.05
	Pine cone	0.010	1.554	0.927	38.054	0.046	0.002	<0.01
	Roots	0.281	1.427	0.929	38.974	0.042	0.002	<0.01
Immature	Total tree	0.036	2.914	0.944	50.821	0.042	0.002	<0.01
	Aboveground	0.041	2.835	0.948	54.753	0.040	0.002	<0.01
	Tree stem	0.021	2.981	0.935	43.438	0.047	0.002	<0.01
	Stem wood	0.021	2.945	0.938	45.006	0.046	0.002	<0.01
	Stem bark	0.001	3.285	0.910	30.366	0.062	0.004	<0.05
	Branch	0.960	1.003	0.936	44.046	0.016	0.000	<0.01
	Needle	0.003	2.945	0.978	135.618	0.026	0.001	<0.01
	Pine cone	2E-11	8.395	0.901	27.297	0.167	0.028	<0.05
	Roots	0.001	3.577	0.913	31.448	0.066	0.004	<0.05
Mature	Total tree	0.785	1.932	0.949	55.366	0.036	0.001	<0.01
	Aboveground	0.465	2.063	0.950	57.171	0.037	0.001	<0.01
	Tree stem	0.493	1.974	0.941	48.147	0.039	0.002	<0.01
	Stem wood	0.536	1.911	0.934	42.307	0.040	0.002	<0.01
	Stem bark	0.010	2.510	0.967	86.847	0.037	0.001	<0.01
	Branch	0.023	2.253	0.970	97.205	0.031	0.001	<0.01
	Needle	0.052	1.988	0.942	48.299	0.039	0.002	<0.01
	Pine cone	4E-05	6.228	0.979	139.558	0.072	0.005	<0.01
	Roots	2.010	0.918	0.906	28.889	0.023	0.001	<0.05

Equations follow the form Y  =  ax^b^ + ε, where a and b are the equation parameters, Y is the biomass of the respective tree component (kg), x is the diameter at breast height (cm), ε is the error term.

a *S.E.E* is the standard error of estimation, *^b^MSR* is the mean square residuals.

Understory biomass was determined using destructive sampling techniques (total harvesting); sampling for shrub layer and herb layer conducted in five 2 m×2 m subplots and 1 m×1 m subplots, respectively. These subplots were randomly selected within each 20 m×30 m plot. Shrubs and herbs were both separated into aboveground and belowground components, and the aboveground components of shrubs were further divided into leaves and branches. Biomass of each component was air dried and subsamples were oven dried at 80°C to constant weight for dry biomass determination.

Subsamples (tissue sample) of *P. tabulaeformis* and understory were collected from each chronosequence stand. Total C stock was obtained by multiplying tissue C concentration by the total dry weight of each component. Biomass of tree and understory were separately calculated and summed to estimated vegetation C density of each stand.

#### Forest floor biomass estimation

Forest floor components were sampled through collecting the entire organic material within five 1 m×1 m subplot randomly chosen in each 20 m×30 m plot. All plant materials collected within each subplot were sorted into three components: undecomposed layer, semi-decomposed layer and full decomposed layer. After the measurements of thickness, subsamples of different components were collected separately, transported to the lab, and oven-dried at 80°C to constant weight. Subsamples were ground and used for C concentration analysis.

The C concentrations of all samples (components of tree, understory, and forest floor) were analyzed by vario Macro Elemental Analyzer (Elementar Analysensysteme GmbH, Germany).

#### Mineral soil sampling and measurement

Soil samples were taken to a depth up to 100 cm with three replicates in each chronosequence stand. At each sampling point, soil samples were extracted from six depths (0–10, 10–20, 20–40, 40–60, 60–80, and 80–100 cm) using a 100 cm^3^ stainless cylinder with. Prior to the measurement of bulk density, samples for each soil layer were sieved through a 2 mm mesh. Bulk density for each soil depth was measured by weighing the whole sample and drying subsamples at 105°C. Soil organic carbon was established by the oil-bath K_2_Cr_2_O_7_ titration method.

### Calculation of C stocks

Forest ecosystem C stocks were calculated as follows: 
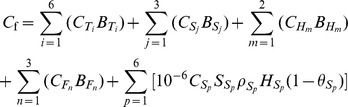


where *C_f_* is the forest ecosystem C stock (Mg C ha^–1^), *i* is the tree tissue type (i.e., stem wood, stem bark, branch, needle, pine cone, and roots), *C_Ti_* is the C concentration of the tree tissue (%), *B_Ti_* is the biomass of the tree tissue (Mg·ha^–1^), *j* is the shrub tissue type (i.e., branch, leaf, and root), *C_Sj_* is the C concentration of the shrub tissue (%), *B_Sj_* is the shrub tissue biomass (Mg·ha^–1^), *m* is the component (i.e., the aboveground and belowground) of herb, *C_Hm_* is the C concentration of the component (%), *B_Hm_* is the component biomass (Mg·ha^–1^), *n* is the forest floor component (i.e., undecomposed layer, semi-decomposed layer and full decomposed layer), *C_Fn_* is the C concentration of the component (%), *B_Fn_* is the biomass of the forest floor component (Mg·ha^–1^), *p* is the layer of mineral soil, *C_Sp_* is the C concentration of the mineral soil (%), *S_Sp_* is the area of the calculated soil (ha), *ρ_Sp_* is the bulk density of the measured soil layer (g·cm^–3^), *H_Sp_* is the depth of the measured soil layer (10 or 20 cm), and 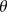 *_Sp_* is the volumetric percentage of fragments >2 mm.

### Statistical analysis

Statistical analysis of data and regression analysis for developing allometric equations were performed using the SPSS software package (ver.17.0; SPSS, Chicago, IL). The difference between the stand mean and variation within that stand was examined by one-way analysis of variance (ANOVA) test.

## Results

### Biomass of ecosystem components

Based upon the power regression equations of tree components (stem wood, stem bark, branch, needle, pine cone, and roots) (Table 2), the biomass were estimated for all *P. tabulaeformis* forests (Table 3). The biomass of each tree component showed that an increasing trend with increasing stand age, with the exception of branch, needle, pine cone and roots. For young and middle-aged stands, the biomass distribution among different tree components was in an order of stem wood >roots >branch >needle >stem bark >pine cone. For the immature stand, however, the average needle biomass was slightly greater than the average branch biomass. In the mature stand, the average stem bark biomass was higher than the average branch biomass. Total tree biomass was 123.8, 189.3, 259.8, and 344.8 Mg·ha^–1^ for the young, middle-aged, immature, and mature stands, respectively, demonstrating a rapid increase from young to mature stands. The stem made the largest contribution to the total biomass regardless of stand age, accounting for 46.9%, 72.2%, 70.6% and 70.7% of total tree biomass for young, middle-aged, immature, and mature stands, respectively. The root to shoot ratios of four stands ranged from 0.1 for the mature stand to 0.2 for the young stand, with an average value of 0.1 across the entire age sequence.

**Table 3:** Biomass of different ecosystem components in *P. tabulaeformis* forests.

Components	Biomass (Mg·ha^–1^)			
	Young	Middle-aged	Immature	Mature
Total tree	123.8±19.2^d^	189.3±8.6^c^	259.8±32.1^b^	344.8±26.2^a^
Aboveground	101.3±15.7^d^	170. 5±7.4^c^	228.8±27.0^b^	314.4±21.0^a^
Tree stem	58.0±9.0^d^	136.7±5.8^c^	183. 3±24.2^b^	243.8±21.5^a^
Stem wood	45.9±7.3^d^	122.8±5.5^c^	163.1±21.1^b^	213.6±19.7^a^
Stem bark	12.1±1.8^c^	13.9±0.4^c^	20.3±3.1^b^	30.1±2.2^a^
Branch	21.8±3.4^b^	17.8±1. 2^bc^	16.6±0.4^c^	29. 6±2.2^a^
Needle	19.3±3.1^bc^	15.0±0.4^c^	20.6±2.7^b^	26.9±2.4^a^
Pine cone	2.2±0.2^b^	1.02±0.1^b^	8.2±4.4^b^	14.2±1.7^a^
Roots	22.4±3.5^b^	18.7±1.3^b^	31.0±5.4^a^	30.5±4.4^a^
Total understory	3.5±0.9^a^	1.8±1.1^b^	2.8±0.5^ab^	2.0±0.6^ab^
Total shrub	0.4±0.4^a^	0.6±0.5^a^	0.8±0.7^a^	0.9±0.5^a^
Shrub foliage	0.0±0.0^b^	0.1±0.1^ab^	0.0±0.0^b^	0.1±0.1^a^
Shrub branch	0.1±0.1^a^	0.3±0.3^a^	0.2±0.2^a^	0.3±0.3^a^
Shrub root	0.2±0.2^a^	0.2±0.2^a^	0.6±0.6^a^	0.4±0.2^a^
Total herb	3.1±1.1^a^	1.2±0.6^b^	2.0±0.3^ab^	1.2±1.2^b^
Aboveground herb	1.0±0.5^a^	0.3±0.2^a^	0.4±0.1^a^	0. 5±0.4^a^
Belowground herb	2.1±0.6^a^	0.9±0.4^b^	1.6±0.2^ab^	0.7±0.7^b^
Forest floor	14.9±2.5^b^	17.5±4.0^b^	20.0±2.7^ab^	23.0±0.9^a^
Undecomposed	6.6±1.0^b^	8.7±2.2^ab^	9.3±1.2^a^	9.7±0.4^a^
Semi-decomposed	4.9±1.0^b^	5.2±1.0^b^	6.5±1.3^ab^	8.2±0.7^a^
Full decomposed	3.4±1.1^a^	3.6±1.2^a^	4.1±2.2^a^	5.1±1.6^a^

Data are presented as the mean value ± the standard deviation (*SD*). Mean values of biomass within a row followed by different lowercase letters are significantly different at *p*<0.05.

The total understory biomass ranged from 1.8 Mg ha^–1^ in the middle-aged stand to 3.5 Mg·ha^–1^ in the young stand. Forest floor biomass increased steady with stand age, ranging from 14.9 to 23.0 Mg·ha^–1^.

### C concentrations and C pools of ecosystem components

#### Tree, understory, and forest floor

**Figure 2:**
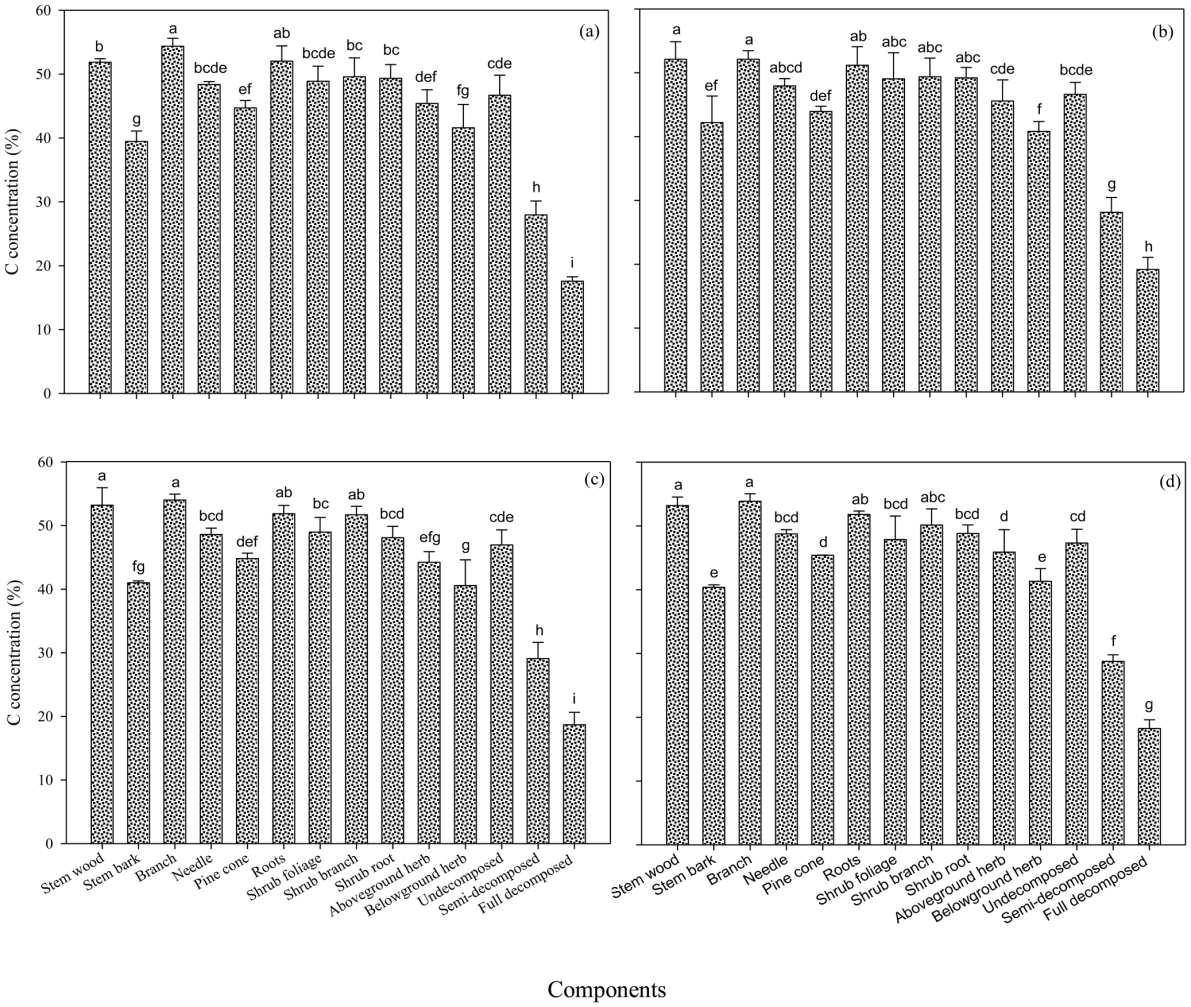
Biomass C concentrations of different components in the young (a), middle-aged (b), immature (c), and mature (d) *P. tabulaeformis* forests. Different lowercase letters indicate a significant difference within-stand (*p*<0.05).

The C concentrations of different components of tree, understory, and forest floor in four age groups were shown in Fig. 2. In general, tree had the highest C concentration while the forest floor had the lowest. The C concentration varied from 39.5% to 54.4% among components within individual trees. The highest and lowest C concentrations in each stand was found to be the branch and stem bark, respectively. The mean C concentration of total tree ranged from 48.2% in the middle-aged stand to 48.9% in the immature stand. Shrub and herb had smaller variation in C concentrations among different components, ranging from 47.9% to 51.7%, and 40.6% to 45.9% of dry biomass, respectively. Within shrub components, higher C concentration was found in the branch than in the other components, with a mean value of 50.2% across all stands. On average, the C concentrations in the shrub foliage had the lowest values compared to the other components. For the immature stand, however, the shrub foliage C concentration was slightly greater than the shrub root C concentration. For all stands, the pattern of C concentrations distribution for the different components of the herb was in an order of aboveground > belowground. Within forest floor components, C concentrations ranged from 17.6% to 47.3% among the *P. tabulaeformis* stands. The C concentrations of forest floor in each stand showed a decreasing trend with the increase in the decomposition level.

The C stock of trees across the age sequence followed a similar pattern as tree biomass due to the steady C concentration for the same component of the differently aged stands (Fig. 2). The aboveground and total tree biomass C increased significantly from 50.8 and 62.4 Mg C ha^−1^ in the young stand to 161.2 and 177.0 Mg C ha^−1^ in the mature stand (Table 4). The contribution of tree stem C to total biomass C of vegetation and forest floor ranged from 41.4% for the young stand to 67.6% for the middle-aged stand. The contribution of pine cone C to the total biomass C was the smallest in each stand.

**Table 4:** C pools of different biomass components in *P. tabulaeformis* forests.

Components	Young		Middle-aged		Immature		Mature	
	(Mg C ha^–1^)(%)		(Mg C ha^–1^)(%)		(Mg C ha^–1^)(%)		(Mg C ha^–1^)(%)	
Total tree	62.4±9.6^d^	90.3±2.4^a^	96.4±4.5^c^	93.3±0.6^a^	133.8±18.7^b^	94.1±0.9^a^	177.0±11.8^a^	95.2±0.5^a^
Aboveground	50.8±7.8^d^	73.4±1.9^a^	86.8±3.8^c^	84.0±0.8^b^	117.8±15.9^b^	82.8±0.5^b^	161.2±10.8^a^	86.8±0.5^b^
Tree stem	28.6±4.4^d^	41.4±1.1^b^	69.9±3.0^c^	67.6±0.8^c^	95.1±12.5^b^	66.9±0.2^c^	125.8±11.1^a^	64.5±3.3^c^
Stem wood	23.8±3.8^d^	34.4±1.1^c^	64.0±2.9^c^	62.0±0.6^d^	86.7±11.2^b^	61.0±0.2^d^	113.6±10.5^a^	58.3±3.3^d^
Stem bark	4.8±0.7^c^	6.9±0.2^f^	5.9±0.2^c^	5.7±0.2^g^	8.3±1.3^b^	5.9±0.1^g^	12.2±0.9^a^	6.2±0.1^f^
Branch	11.8±1.9^b^	17.1±0.6^d^	9.3±0.6^c^	9.0±0.2^e^	9.0±0. 2^c^	6.4±0.8^fg^	15.9±1.2^a^	8.2±0.2^f^
Needle	9.4±1.5^b^	13.5±0.5^e^	7.2±0.2^c^	6.9±0.2^f^	10.0±1.3^b^	7.1±0.0^f^	13. 1±1.1^a^	6.7±0.3^ef^
Pine cone	1.0±0.1^b^	1.4±0.2^hi^	0.4±0.0^b^	0.4±0.0^j^	3.7±2.0^b^	2.5±1.0^i^	6.440±0.8^a^	3.5±0.6^g^
Roots	11.7±1.8^b^	16.9±0.6^d^	9.6±0.7^b^	9.3±0.2^e^	16.1±2.8^a^	11.3±0.5^e^	15.8±2.3^a^	8.1±1.0^e^
Total understory	1.5±0.4^a^	2.2±0.5^h^	0.8±0.5^b^	0.8±0.6^ij^	1.2±0.3^ab^	0.9±0.1^jk^	0.9±0.2^ab^	0.5±0.1^i^
Total shrub	0.2±0.2^a^	0.3±0.2^i^	0.3±0.3^a^	0.3±0.3^j^	0.4±0.3^a^	0.3±0.2^kl^	0.4±0.3^a^	0.2±0.1^i^
Shrub foliage	0.0±0.0^b^	0.0±0.0^j^	0.0±0.0^ab^	0.0±0.0^j^	0.0±0.0^b^	0.0±0.0^l^	0.1±0.0^a^	0.0±0.0^i^
Shrub branch	0.1±0.1^a^	0.1±0.1^j^	0.1±0.2^a^	0.2±0.2^j^	0.1±0.1^a^	0.1±0.1^l^	0.2±0.1^a^	0.1±0.1^i^
Shrub root	0.1±0.1^a^	0.2±0.2^j^	0.1±0.1^a^	0.1±0.1^j^	0.3±0.3^a^	0.2±0.2^kl^	0.2±0.1^a^	0.1±0.1^i^
Total herb	1.3±0.5^a^	1.9±0.7^hi^	0.5±0.3^b^	0.5±0.3^j^	0.8±0.1^ab^	0.6±0.1^jkl^	0.5±0.5^b^	0.3±0.3^i^
Aboveground herb	0.4±0.2^a^	0.6±0.3^hi^	0.1±0.1^a^	0.2±0.1^j^	0.2±0.1^a^	0.1±0.0^kl^	0.2±0. 2^a^	0.1±0.1^i^
Belowground herb	0.9±0.2^a^	1.3±0.4^hi^	0.4±0.2^b^	0.4±0.2^j^	0.6±0.1^ab^	0.5±0.1^kl^	0.3±0.3^b^	0.2±0.2^i^
Forest floor	5.1±0.8^c^	7.5±2.2^f^	6.2±1.4^bc^	6.0±1.1^g^	7.1±0.5^ab^	5.1±0.9^h^	7.9±0.2^a^	4.1±0.3^g^
Undecomposed	3.1±0.4^b^	4.6±1.3^g^	4.0±1.0^ab^	3.9±0.8^h^	4.4±0.6^a^	3.1±0.7^i^	4.6±0.2^a^	2.4±0.2^gh^
Semi-decomposed	1.4±0.3^b^	2.0±0.6^h^	1.5±0.3^b^	1.4±0.2^i^	1.9±0.4^ab^	1.4±0.4^j^	2.3±0.2^a^	1.2±0.1^h^
Full decomposed	0.6±0.2^a^	0.9±0.4^hi^	0.7±0.2^a^	0.7±0.2^ij^	0.8±0.4^a^	0.6±0.3^kl^	0.9±0.3^a^	0.5±0.2^i^

Data are presented as the mean value ± the standard deviation (*SD*). Mean values of C stocks within a row and mean percentages of C stocks of biomass components within a column followed by different lowercase letters are both significantly different at *p*<0.05.

The predicted C stocks stored in different components of total understory (shrub and herb) were generally higher than the observed C stocks if using the C conversion factor (0.5). The observed C stocks of total understory in the *P. tabulaeformis* stands varied from 0.8 Mg C ha^–1^ in the middle-aged stand to 1.5 Mg C ha^–1^ in the young stand, most of which came from herb layer (Table 4). The C stored in the total herb for the young, middle-aged, immature, and mature stands accounted for 1.9%, 0.5%, 0.6%, and 0.3% of the total biomass C pools, respectively.

The observed C stocks in forest floor increased steadily with stand age, from 5.1 Mg C ha^–1^ in the young stand to 7.9 Mg C ha^–1^ in the mature stand (Table 4). The C stored in the forest floor for the young, middle-aged, immature, and mature stands accounted for 7.5%, 6.0%, 5.1%, and 4.1% of the total biomass C pools, respectively.

#### Mineral soil

**Figure 3:**
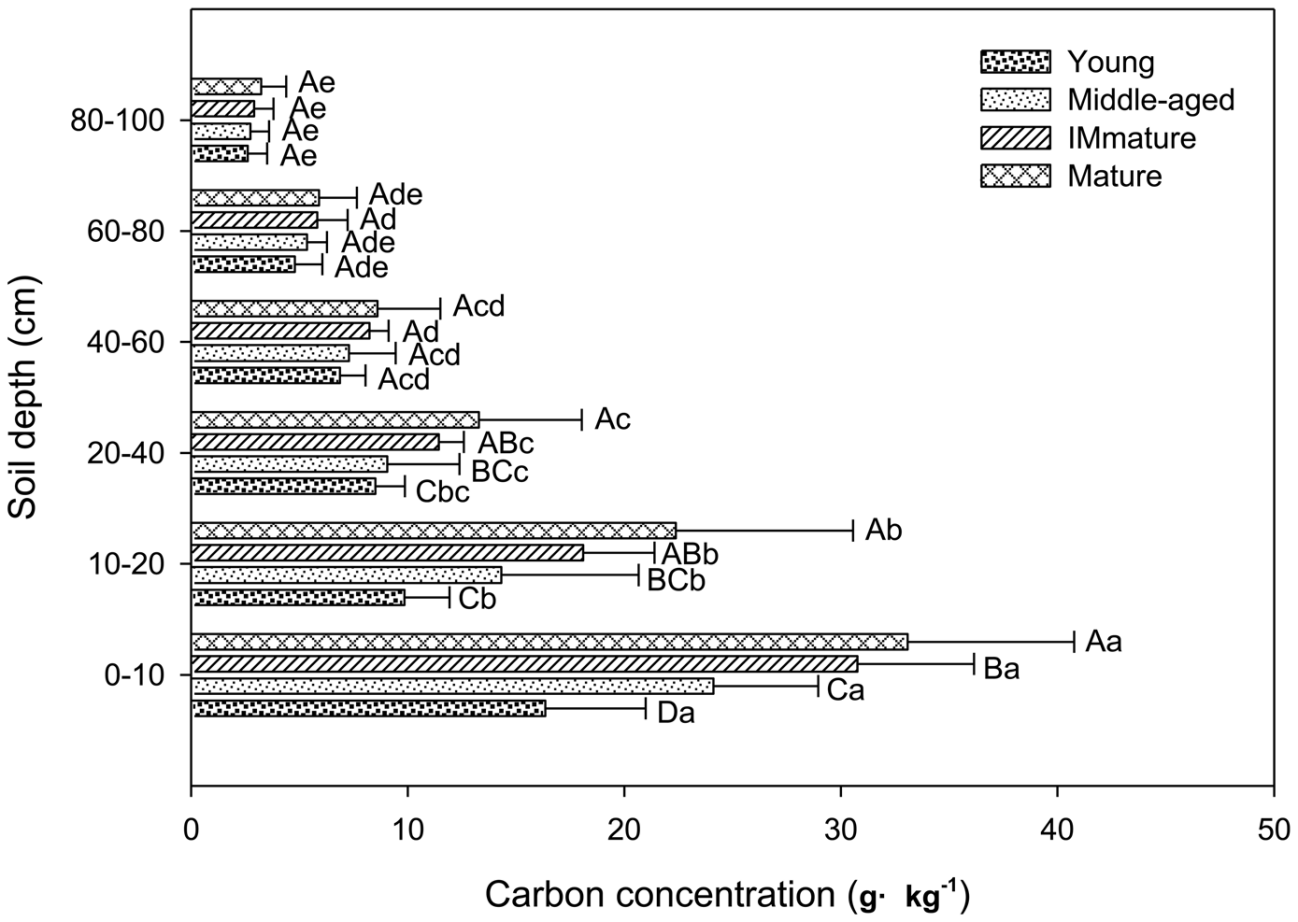
MSOC concentrations at different soil depths in the *P. tabulaeformis* forests. Different uppercase letters indicate a significant difference between age groups in the same depth (*p*<0.05), different lowercase letters indicate a significant difference between different soil depths within-stand (*p*<0.05). Error bars standard deviation (*SD*).

**Figure 4:**
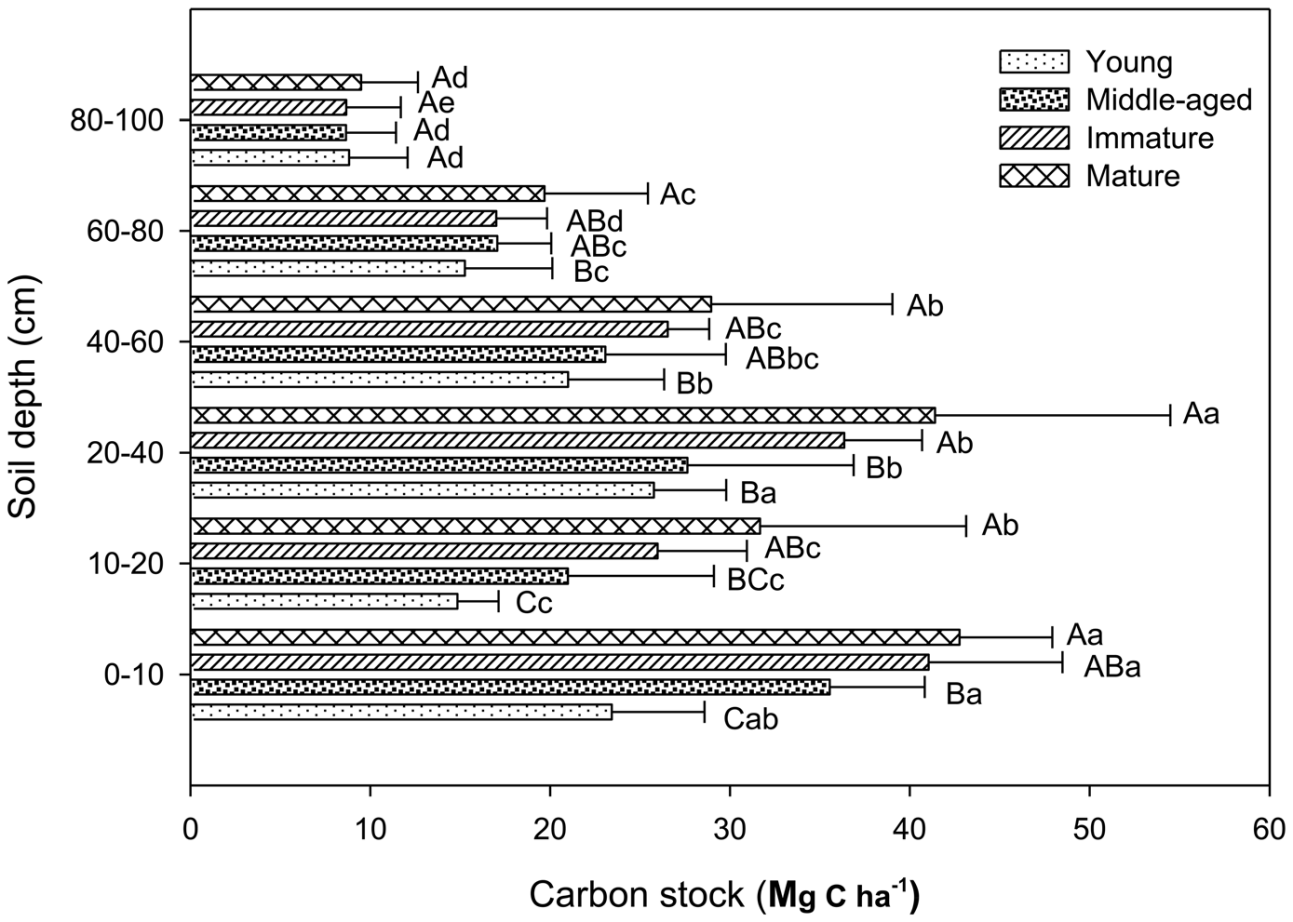
MSOC stocks at different soil depths in the *P. tabulaeformis* forests. Different uppercase letters indicate a significant difference between age groups at the same horizon (*p*<0.05), different lowercase letters indicate a significant difference between different soil depths within-stand (*p*<0.05). Error bars standard deviation (*SD*).

Mineral soil organic carbon (MSOC) concentration at all soil depths increased with stand age. At depth of 0–10, 10–20, 20–40, 40–60, 60–80, and 80–100 cm, MSOC concentrations of the young stand were 16.4, 9.9, 8.5, 6.9, 4.8, and 2.6 g·kg^−1^, respectively, and MSOC concentrations of the mature stand were 33.1, 22.4, 13.3, 8.6, 5.9, and 3.2 g·kg^−1^, respectively. The MSOC concentration decreased significantly with increasing soil depth in all stands (Fig.3). However, it increased significantly in the same depth cross the chronosequence. The observed C stocks in total mineral soil continuously increased with stand age, from 109.1 Mg C ha^–1^ in the young stand to 174.0 Mg C ha^–1^ in the mature stand. The MSOC stock between the 0–40 cm was greater than that in deeper soil (Fig.4), accounting for 58.7%, 63.3%, 66.4%, and 66.6% of entire soil profile (0–100 cm) in the young, middle-aged, immature, and mature stands, respectively.

#### Forest ecosystem

The C stocks of the tree, shrub, forest floor, mineral soil, and total ecosystem increased with increasing stand age, but for herb, C stock was the highest in the young stand (Table 5).

**Table 5:** C pools of ecosystem components in *P. tabulaeformis* forests.

Components	C stock (Mg C ha^–1^)			
	Young	Middle-aged	Immature	Mature
Tree	62.4 ±5.6	96.4±2.6	133.8 ±10.8	177.0±6.8
Shrub	0.2±0.1	0.3±0.2	0.4±0.2	0.4±0.1
Herb	1.3±0.3	0.5±0.1	0.8±0.1	0.5±0.3
Forest floor	5.1±0.5	6.2±0.8	7.1±0.3	7.9±0.1
Mineral soil	109.1±5.8	133.0±10.6	155.6±4.2	174.0±11.7
Ecosystem	178.1±7.7	236.3±18.4	297.7±11.0	359.8±21.0

Data are presented as the mean value ± the standard error (*SE*).

**Figure 5:**
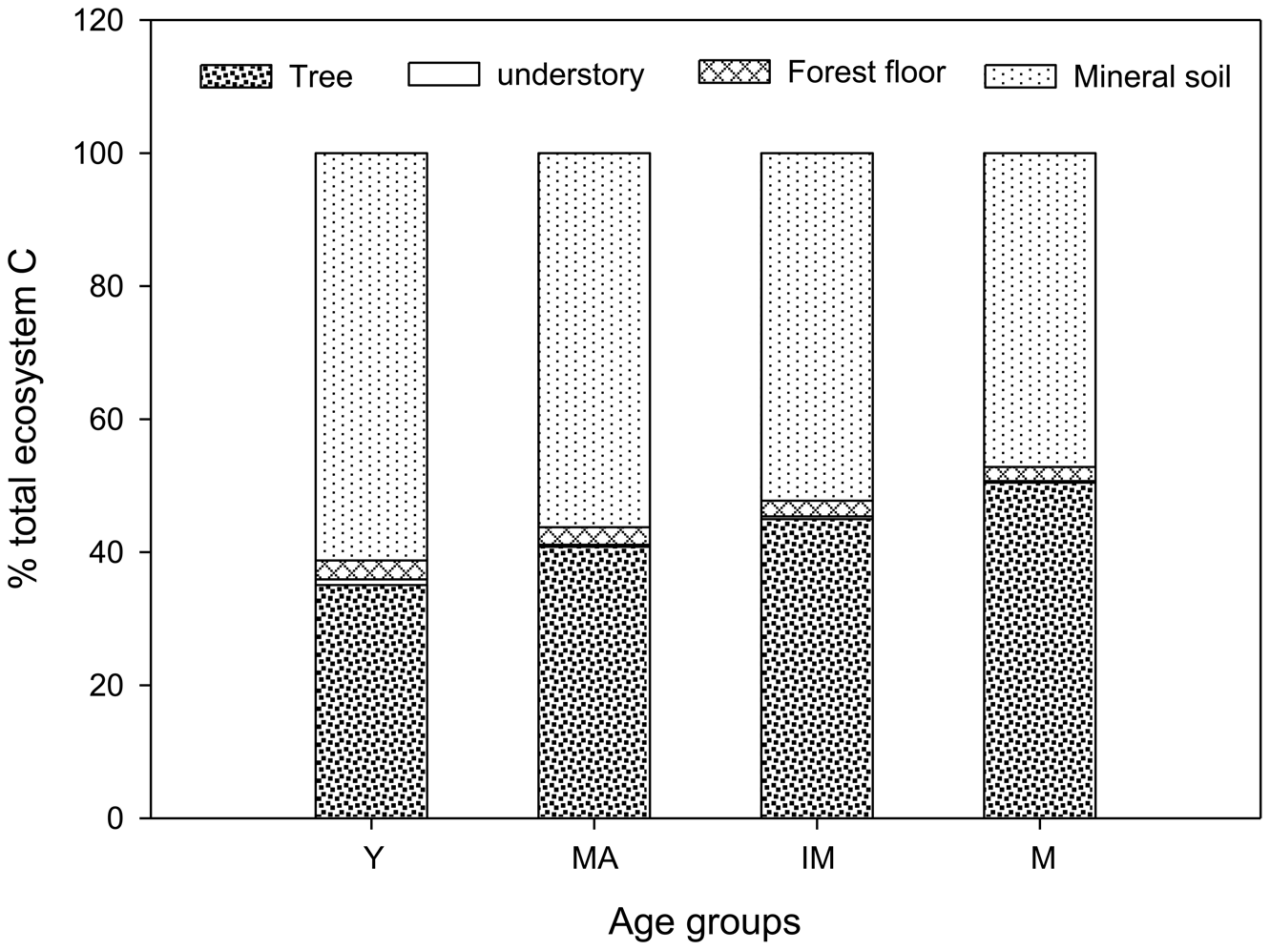
Percentage contribution of C pool in individual components of ecosystem in the young (Y), middle-aged (MA), immature (IM), and mature (M) *P. tabulaeformis* forests.

Tree and mineral soil were the two largest contributors to the total ecosystem C pool in our age sequence stands (Fig.5). The contribution of tree biomass C ranged from 35.1% for the young stand to 49.2% for the mature stand, with an average value of 42.5% in this chronosequence study. Mineral soil was the dominant C pool, representing 61.3% of the total ecosystem in the young stand, whereas the contribution of MSOC decreased only to 48.4% in the mature stand. Forest floor C represented 2.2% to 2.8% of the total ecosystem C pool. The total understory contributed little to the forest ecosystem C pool.

## Discussion

### Biomass

#### Tree

Our estimates of *P. tabulaeformis* biomass across the chronosequence demonstrated that tree biomass increased with the age of stands. This pattern was in agreement with many previous reports on other pine forests [16], [27], [29], [39], confirming that stand age is an important variable for accurately estimating biomass of different components in pine forests. Meanwhile, our results revealed *P. tabulaeformis* biomass was greater than other pine species across the same chronosequence studied in China (Table 6). For example, Gower et al. [40] reported that the average value of total tree biomass of temperate pines is 120.5 Mg·ha^–1^, which is smaller than the average value we found. Cao et al. [29] and Li et al. [41] studied the biomass accumulation across different stages of stand development of pine forests, and the result is also smaller than what we reported here. The greater biomass revealed in our study indicates that *P. tabulaeformis* forests in Liaoheyuan have a greater potential to accumulate biomass. These differences may be affected by the stand age and origin.

**Table 6:** Comparison of biomass between *P. tabulaeformis* and published results (FRSC, 1994) of pine species.

Pine species	Biomass (Mg·ha^–1^)				
	Young	Middle-aged	Immature	Mature	Fang's data*
*P.sylvestris var. mongolica*	40.42	93.01	113.37	122.9	49.3
*P.tabulaefomis*	62.71	81.92	82.05	93.76	88.7
*P.armandii, P. densata*	76.71	134.22	169.25	191.87	71.8
*P.koraiensis*	74.91	171.89	214.46	245.52	65.4
*P.massoniana*	70.53	126.31	322.20	319.49	81.1
*P.yunnaensis, P. khasya*	58.79	108.87	197.11	219.99	nd*

^*^nd indicates that the results were not determined at these sites.

The patterns of biomass distribution among various components of the tree were the same for both young and middle-aged stands, average biomass decreasing in an order of stem wood > roots > branch > needle > stem bark > pine cone (Table 3). Similar patterns were observed in an age sequence of *P. densiflora* stands [19] and *lacebark pine* plantation forests [41]. However, inconsistent with other reports, we found that branch biomass was lower than needle biomass and stem bark biomass in the immature and mature stands, which is likely due to the large within-stand variability.

Previous studies have reported that the proportion of stem to total tree biomass varied significantly among stands of different ages [39]. The ratios in our study were 46.9%, 72.2%, 70.6% and 70.7% for young, middle-aged, immature, and mature stands, respectively. Therefore, total tree biomass may be considerably underestimated by forest inventories that are normally limited to stem biomass. At the same time, root biomass accounted for a large proportion of the total tree biomass, highlighting the importance of roots in the total biomass estimation of *P. tabulaeformis* forests.

#### Understory and forest floor

Only a few studies have measured understory and forest floor biomass in *P. tabulaeformis* natural forests in Northern China. In our study, the understory biomass ranged from 1.8 Mg·ha^–1^ in the middle-aged stand to 3.5 Mg·ha^–1^ in the young stand. These numbers were in agreement with other previously reported values of 1.61–3.76 Mg·ha^–1^ [39] and 0.87–3.55 Mg·ha^–1^ [29]. However, our results were lower than Li et al. [41] whose estimates of understory biomass were 6.17 and 20.25 Mg·ha^–1^ in 16- and 35-year old *lacebark pine* stands, respectively. We also found a sigmoid pattern of changes in understory biomass across the chronosequence. Understory biomass might not be responsive to stand age. It may depend more on forest management, stand-specific canopy, and soil conditions, which affect light, water, and nutrient availability for the development of understory.

Forest floor biomass, however, did increase steadily from young to mature stands along the chronosequence, ranging from 14.9 to 23.0 Mg·ha^–1^. This pattern was in accordance with the results from Li et al. [41], who reported that the forest floor biomass of secondary *lacebark pine* increases with stand age.

#### C concentration

A factor of 0.5 of the C concentration has been commonly used for C conversion from biomass [42]. Recent studies showed that the C concentration of tree components or tree species might be either above or below 0.5 [29], [39], [41], [43]. In our study, C concentrations fell within a range of 39.5% to 54.4% of dry biomass, and the mean C concentration of total tree ranged from 48.2% in the middle-aged stand to 48.9% in the immature stand. Overall, the C concentration of shrub layer (47.9–51.7%) was higher than herb layer (40.6–45.9%), similar to previous results reported for various pine species [29], [39], [41]. Within forest floor, higher C concentration was found in the undecomposed layer than that in other components, with a mean value of 46.9% across the chronosequence stands. We found that the C concentrations of ecosystem components changed significantly in each chronosequence stand (Fig. 2). This indicates that the use of a component-specific C concentration to estimate the forest C pool is more appropriate than a fixed conversion factor (0.5). We also found that the C concentrations of ecosystem components in *P. tabulaeformis* forests did not change significantly with stand age (*p*>0.05), which is similar to the results reported by Cao et al. [29] and Uri et al. [44]. We suggest that a component-specific C concentration value is suitable for estimating forest C pool among all stands.

#### Carbon storage

The estimated total tree biomass increase steadily with stand age, from 62.4 Mg C ha^–1^ in the young stand to 177.0 Mg C ha^–1^ in the mature stand. The pattern of changes in total tree biomass among stand ages is the same between our study and previous studies. For example, Li et al. [41] reported the total tree C stock of *lacebark pine* increased from 23.6 to 148.8 Mg C ha^–1^ when stand ages increased from 16 to 68 years old. Total tree biomass estimates often vary among pine forests [19], [29], [45], [46], which is likely due to the differences in the stand density and nutrients of forests.

The pattern of understory C stock changes with stand ages was different in our study than in other coniferous chronosequence studies (Table 7). Previous research has shown that the C stock of the understory decreased with the increasing stand age [21], [29], but the C stock of the understory in our study was not significantly related to stand age. This may be explained by the increasing competition for light and nutrients with increasing stand age. Interestingly, we found total shrub C increased with stand age, similar to previous result reported for conifer [43]. However, herbs dominated understory C pool in our chronosequence of *P. tabulaeformis* stands, and the contribution of herb C to the total understory C varied from 55.3% to 87.6%, resulting in a sigmoid pattern of changes in total understory C pool along our chronosequence.

**Table 7:** Comparison of C storage between *P. tabulaeformis* and published results of pine species.

Pine species	Age (years)	C stock (Mg C ha^–1^)					
		Tree	Understory	Forest floor	Mineral soil	Ecosystem	Reference
*P. strobus*	16–114	24.5–174.4	nd*	nd*	2.4–17.1	33.6–239.4	[45]
*P. strobus*	2–65	0.2–103.4	2.1–3.6	0.8–12.1	33.9–39.1	40.2–156.1	[16]
*P. elliottii*	>14	31.1	8.1	2.2	65.4	104.07	[43]
*P. massoniana*	>14	35.2	19.5	3.0	84.3	142.0	[43]
*P. densiflora*	10–71	9.3–104.9	nd*	7.0–11.2	2.5–84.7	18.8–201.4	[19]
*P. koraiensis*	8–51	0.8–122.3	0.6–1.1	3.14–6.1	32.9–37.6	42.2–162.7	[39]
*P. kesiya*	65–80	222.9	0.4	2.2	58.7	283.1	[46]
*P. tabulaeformis*	25–105	20.7–103.8	1.6–3.7	5.9–16.6	53.7–85.6	84.1–207.5	[29]
*P. bungeana*	16–68	23.6–148.8	2.6–13.6	1.1–3.5	66.1–74.4	93.4–240.3	[41]
*P. tabulaeformis*	< 80	62.4–177.0	0.8–1.5	5.1–7.9	109.1–174.0	178.1–359.8	Our results

^*^nd indicates that the results were not determined at these sites.

We found that the total C stock of forest floor increased with stand age. This pattern is consistent with some of the coniferous chronosequence studies [15], [47], but not with others [16], [39], suggesting a large within-stand variability. The previous studies has been reported to be highly impressionable to disturbances and variations in stand treatment, litter input, and decomposition rate [15], [16], [21]. By comparing the trends, it was clear that each component of forest floor C stock increases as the stand age increases and this was due to the increase of litter input and slow decomposition. We also found that the C stock of the forest floor in each stand is greater than that of the total understory, indicating that forest floor should be considered as an important component in total C stock calculation.

The effect of stand age on MSOC is debatable. Among those chronosequence-based studies listed in Table 7, there exist great deals of controversies regarding whether or not MSOC stock could respond to stand ages [48], [49]. Some studies reported no significant increase in mineral soil C stocks with stand age [16], [48], [50], whereas other studies indicate an increasing soil C stocks in the early decades after afforestation [15], [45], [49]. This discrepancy may be due in part to how much other influencing factors, such as climate, soil properties, and forest type, could overshadow the effect of stand age [16]. In our study, we observed soil C stocks increased with stand age, joining with some of the most recent reports to support that conifer forest MSOC does increase with stand age, possibly due to a larger accumulation of organic matter in older stands [29], [41]. Meanwhile, the C stocks of mineral soil in our study were much higher than most others reported (Table 7). These differences may result from the different depths of soil sampling in the profile.

In terms of vertical distribution of MSOC, our data revealed that a large quantity of the MSOC was sequestrated in upper 20 cm of the mineral soil horizon in all stands, indicating that higher amounts of MSOC were stored in the surface layer. Approximately 58.7% to 66.6% of MSOC in the 0–100 cm range of soil was stored in the 0–40 cm range, where soils can be disturbed by human disturbances. Hence, research the spatial variability of MSOC [51], and protection of the topsoil from disturbances is important for C sequestration.

Another important finding through our study was that although the aboveground (sum of vegetation, and forest floor) and belowground (mineral soil) C stocks both increased with stand ages, the contribution of the MSOC stock to total ecosystem C stock decreased gradually from 61.3% in the young stand to 48.4% in the mature stand. This pattern did not occur in all forests as the rates of aboveground and belowground C accumulation differed among tree species during stand development [16], [29], [39]. Considering that the aboveground C accumulation would ultimately exceed the mineral soil C accumulation in mature stand, we suggest that aboveground tree biomass may make major contribution to total ecosystem C over time.

## Conclusions

The biomass of each tree component could be predicted from an allometric equation using *DBH* as the independent variable. Biomass of tree, shrub, and forest floor increased with stand age, particularly the biomass within tree stem which comprised the main proportion of the aboveground and total tree biomass increased with increasing stand age. The highest C concentration was found in tree branch while the lowest C concentration was observed in the forest floor. The use of component-specific C concentration values other than a fixed factor of 0.5 to convert biomass to C stock is needed in order to more accurately estimate the forest C pool. Belowground components contribute a great deal to the total C accumulation, but aboveground tree biomass becomes increasingly important as stands age, indicating the necessity of considering the entire development stages of a forest in estimating its ecosystem-level C stock. The C sequestration potential of *P. tabulaeformis* forests in northern China is greater than previously estimated, so its importance in combating global warming deserves more attention and further studies.

These findings given by our study will improve our understanding of C stocks and dynamics in *P. tabulaeformis* forests and can be used in forest management activities to enhance C sequestration. Due to the lack of over-mature stands in our study, the results do not represent the whole growth pattern of a stand development. Thus, further evidence of the effect of stand age on ecosystem C pool development will be needed from a whole chronosequence studies.
