# Correction: Patterns of Biomass and Carbon Distribution across a Chronosequence of Chinese Pine (*Pinus tabulaeformis*) Forests

**DOI:** 10.1371/journal.pone.0104464

**Published:** 2014-07-29

**Authors:** The PLOS ONE Staff

There are errors in Table 1. The publisher apologizes for these errors. The authors have provided a corrected version of Table 1 here.

**Table 1:** Characteristics of stands of *P. tabulaeformis* natural secondary forests.

Age groups	Stand age (years)	Location (lat, long)	Elevation (m)	Slope degree (°)	Stand density (stems·ha^–1^)	Bulk density* (g·cm^–3^)	*DBH* * (cm)	Height* (m)	Basal area of *DBH* (m^2^·ha^–1^)	Stand volume** (m^3^·ha^–1^)
Young	<30	41°18′N, 118°30′E	1097±6	27±2	2567±112	1.5±0.1	11.0±5.1	9.2±2.0	29.5	270.2
		41°19′N, 118°33′E	1094±5	29±2	2334±35	1.7±0.2	11.8±5.5	10.2±2.1	31.0	283.4
		41°18′N, 118°31′E	1058±10	30±1	1900±93	1.4±0.1	11.0±6.1	9.6±2.4	23.5	215.3
Middle-aged	31–50	41°17′N, 118°31′E	1008±8	30±2	1034±9	1.6±0.1	18.4±7.5	14.9±4.2	32.1	307.2
		41°18′N, 118°32′E	982±3	23±1	1050±28	1.6±0.1	18.5±5.2	15.8±2.0	30.4	291.5
		41°20′N, 118°34′E	985±2	25±1	1034±41	1.5±0.1	17.2±4.6	13.2±2.6	25.8	247.6
Immature	51–60	41°15′N, 118°31′E	1011±14	31±2	884±17	1.6±0.1	18.9±7.7	15.9±4.2	28.9	276.7
		41°17′N, 118°30′E	993±15	30±3	850±53	1.5±0.1	20.4±8.4	17.5±4.4	32.5	263.9
		41°19′N, 118°31′E	1018±9	31±1	867±22	1.4±0.1	20.0±5.3	17.0±2.1	29.2	237.3
Mature	61–80	41°18′N, 118°28′E	1066±18	29±2	917±80	1.6±0.1	23.3±7.1	20.9±3.7	42.5	419.3
		41°16′N, 118°31′E	1080±15	31±2	934±106	1.5±0.2	22.9±10.2	20.1±4.3	45.8	452.4
		41°20′N, 118°30′E	1089±10	23±1	717±37	1.5±0.2	23.1±12.5	19.8±4.9	38.5	379.9

^*^: stand mean ± within-stand *S.D.*

^**^: Stand volume (*M*) and sample tree volume (*V*) was calculated by the following formula (1) and (2), respectively.
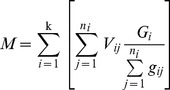
(1)

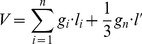
(2) Where *M* is the stand volume (m^3^·ha^–1^), *n_i_* is the number of *i*–th class of the sample tree, *k* is graded series (*i  = * 1, 2, …, *k*), *G_i_* is the *i*–th class of basal area of *DBH* (m^2^·ha^–1^), *V_ij_* and *g_ij_* are the volume (m^3^) and basal area of *DBH* (m^2^) of *j*–th sample tree in *i*–th class, respectively. *V* is the sample tree volume (m^3^), *g_i_* is the central basal area (m^2^) of the *i*–th log, *l_i_* is the length (m) of the *i*–th log, *g_n_* is the last basal area (m^2^) at the top of tree, *l*′ is the length (m) between the last basal area and the top of tree, *n* is the total number of logs.
